# Isolated superior mesenteric artery dissection

**DOI:** 10.4103/0971-3026.63047

**Published:** 2010-05

**Authors:** Palle Lalitha, Balaji Reddy

**Affiliations:** Department of Radiology, Focus Diagnostic Center, Punjagutta, Hyderabad, India

**Keywords:** Abdominal pain, artery of Drummond, superior mesenteric artery dissection

## Abstract

Isolated superior mesenteric artery (SMA) dissection without involvement of the aorta and the SMA origin is unusual. We present a case of an elderly gentleman who had chronic abdominal pain, worse after meals. CT angiography, performed on a 64-slice CT scanner, revealed SMA dissection with a thrombus. A large artery of Drummond was also seen. The patient was managed conservatively.

## Introduction

Chronic pain abdomen(worse after meals) due to mesenteric ischemia caused by spontaneous superior mesenteric artery (SMA) dissection, with no other abnormality or aortic dissection, is unusual. There have been 56 cases of isolated SMA dissection reported in literature.[1] We report a case of spontaneous, isolated SMA dissection with thrombosis, not involving the aorta or the SMA origin, which was treated conservatively with oral anticoagulants.

## Case Report

**Figure 1:**
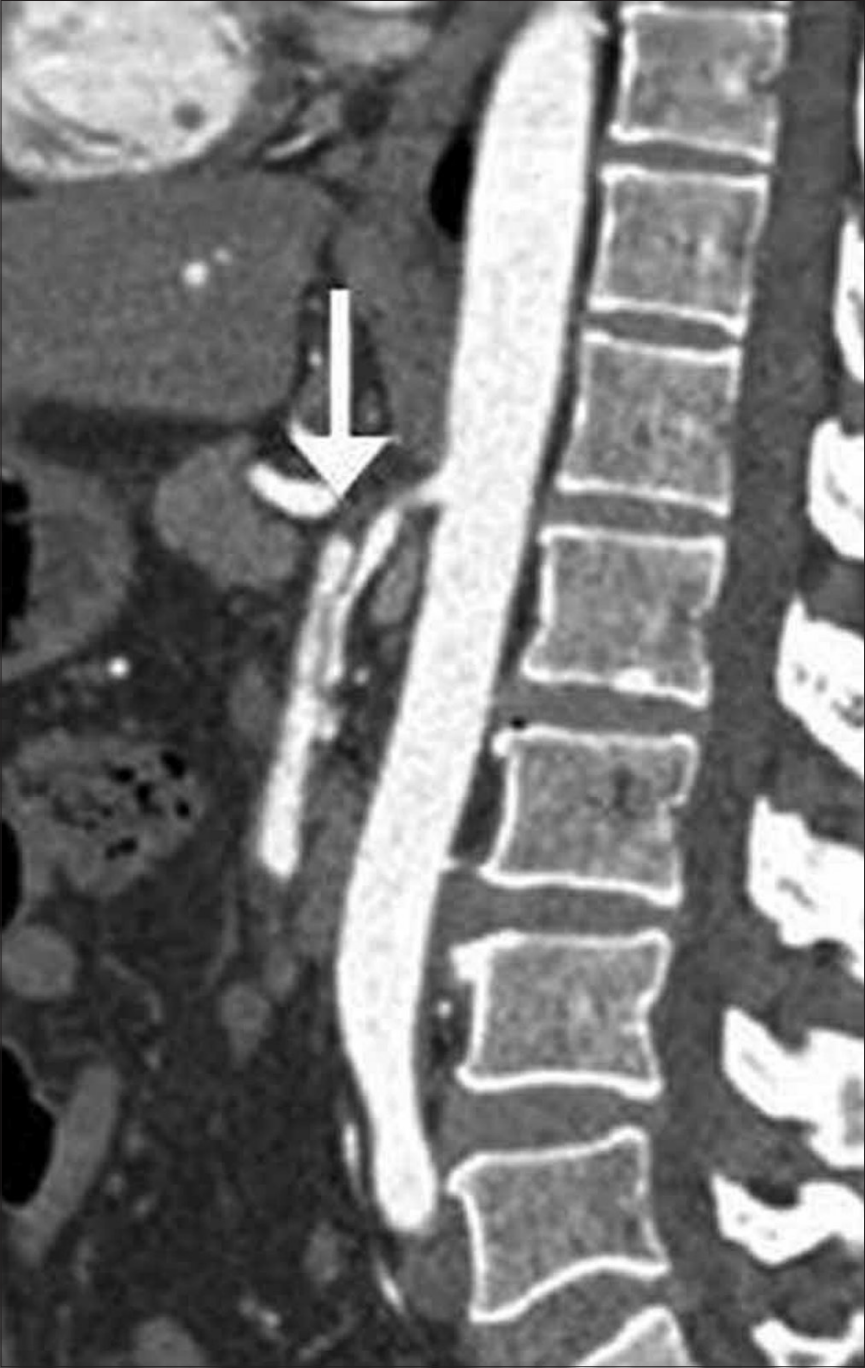
Sagittal, contrast-enhanced, arterial phase, maximum intensity projection CT scan shows a dissection flap (arrow) in the superior mesenteric artery

An elderly gentleman aged 65 years presented with recurrent episodes of abdominal pain for one week; the pain was especially worse after meals. He had no other significant medical history. He did not have diabetes, hypertension, or recent trauma. On physical examination, minimal epigastric tenderness was elicited on deep palpation. His laboratory investigations revealed no significant abnormality. USG of the abdomen revealed no abnormality. With a strong clinical suspicion of mesenteric ischemia, the patient was referred for a CT mesenteric angiogram. Abdominal CT with angiography was performed on a 64-slice CT (Seimens Somatom). This revealed an increased SMA diameter, with an eccentric thrombus in the proximal SMA, extending for a length of 1 cm [Figure 1].

**Figure 2:**
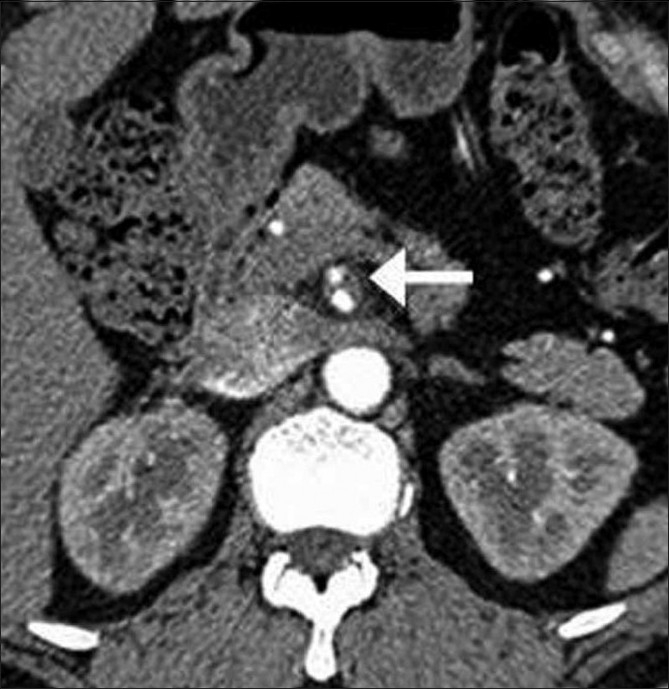
Axial, arterial-phase, contrast-enhanced, CT scan shows superior mesenteric artery dissection (arrow) with patent true and false lumens

**Figure 3:**
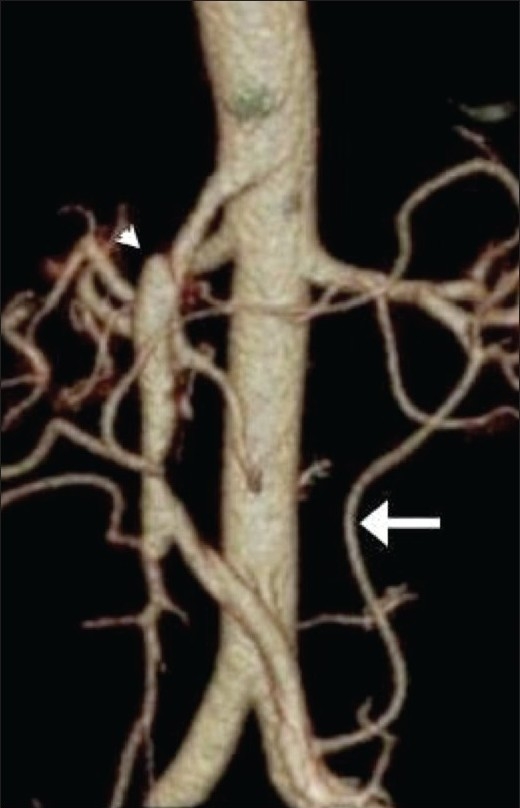
Frontal, volume rendered 3DCT reconstruction demonstrates an enlarged superior mesenteric artery lumen (arrowhead) with dissection and the artery of Drummond (arrow)

Distal to the thrombus, an intimal flap was identified [Figure 2]. The SMA origin was normal. The dissection extended up to the terminal SMA. No thrombosis was seen in either the false or the true lumen. The artery of Drummond was seen supplying the false lumen [Figure 3]. Signs of bowel ischemia, such as bowel wall thickening, abnormal enhancement, or ascites, were not present.

**Figure 4:**
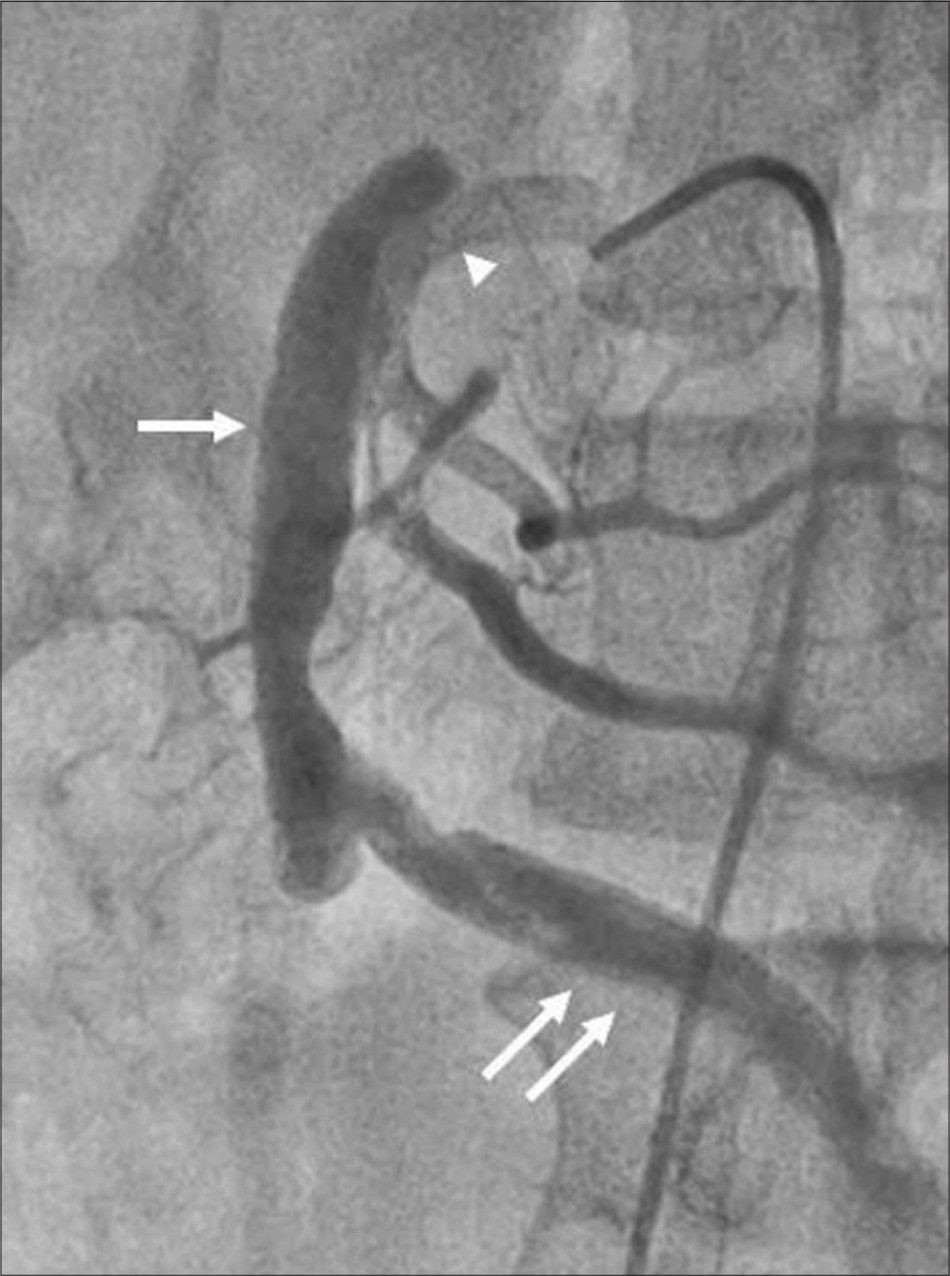
Digital subtraction angiogram shows retrograde filling of the false lumen (single arrow) via the artery of Drummond (double arrow). Minimal contrast is noted in the true lumen (arrowhead)

The aorta and the celiac and inferior mesenteric arteries were normal in caliber, with no thrombus or dissection. No atheromatous changes in the form of wall thickening, plaques, or wall calcification were seen in the aorta. There was no wall irregularity. Conventional SMA angiography was performed, which revealed similar findings. On inferior mesenteric artery injection, the false lumen showed retrograde filling via the artery of Drummond [Figure 4].

In view of the large size of the artery of Drummond and the absence of bowel compromise, intervention was deferred and the patient was put on conservative management with oral anticoagulants. Follow-up CT angiography after one month showed no progression. The patient was also symptom-free. The exact etiology of isolated SMA dissection in this patient could not be identified.

## Discussion

SMA dissection is an uncommon cause of chronic abdominal pain, the pain being especially worse after a meal. In 1947, Bauersfeld reported the first case.[1] Since then, 56 cases have been reported. Isolated SMA dissection occurs more commonly in males. Cases have been reported in the age-group of 41-71 years.[1] Abdominal pain is the commonest symptom and is probably due to dissection or bowel ischemia. Pain may be accompanied by nausea. However, seven of the reported patients did not have any pain in the abdomen and the dissection was detected only incidentally;[2–5] the diagnosis of dissection being confirmed on imaging.

In the hypothesis proposed by Solis *et al*. dissection was thought to be caused by stress on the wall of the artery at the inferior pancreatic edge.[6] Although many causes like atherosclerosis, fibrodysplasia, connective tissue disorders, and trauma have been proposed, the definite etiology is not known.[7] This was true in our patient as well, where the exact cause of SMA dissection could not be ascertained. In view of the age of our patient and the absence of other known causes, the most likely possibility was perhaps atherosclerosis.

Although dissection can be seen with the help of Doppler USG, CT angiography is a more appropriate investigation for the demonstration of the dissection flap, the thrombus (if any), and the true and false lumens. It may not be possible to view the intimal flap in all cases, and an increased diameter of the SMA with increased attenuation of the surrounding fat may be the only clues to the presence of dissection.[7] Dissection must be included in the differential diagnosis in cases of luminal thrombosis in an enlarged SMA with no definite intimal flap. In our case, excellent demonstration of the proximal thrombus, the site of dissection, the false and true lumens, and the artery of Drummond was possible on our 64-slice multidetector CT. The patient did not have any visible bowel wall pathology at the time of examination.

There is no clear, rigid protocol for the treatment of SMA dissection. Surgery, stents, and conservative management are among the treatment options available. Patients put on conservative management should be followed up to look for signs of worsening mesenteric ischemia. Follow-up CT performed after one month in our patient did not reveal any changes as compared to the previous CT.

## Conclusion

We report a case of isolated spontaneous SMA dissection without aortic involvement in an elderly gentleman who had presented with chronic abdominal pain that was especially worse after food intake. This patient was managed conservatively. The diagnosis could be made accurately with help of a 64-slice multidetector CT. Patients on conservative management should be on close follow-up to look for signs of worsening mesenteric ischemia.
